# Effects of acetylsalicylic acid and ultrasound elastography on testicular torsion in rats

**DOI:** 10.55730/1300-0144.5729

**Published:** 2023-10-12

**Authors:** Ezgi GÜN SOYTÜRK, Ünal BIÇAKCI, Serdar ASLAN, Tolga GÜVENÇ, Meltem CEYHAN BİLGİCİ

**Affiliations:** 1 Division of Pediatric Urology, Department of Pediatric Surgery, Samsun Training and Research Hospital, Samsun, Turkiye; 2 Division of Pediatric Urology, Department of Pediatric Surgery, Faculty of Medicine, Ondokuz Mayıs University, Samsun, Turkiye; 3 Department of Radiology, Faculty of Medicine, Giresun University, Giresun, Turkiye; 4 Department of Veterinary Pathology, Faculty of Veterinary, Ondokuz Mayıs University, Samsun, Turkiye; 5 Department of Radiology, Faculty of Medicine, Ondokuz Mayıs University, Samsun, Turkiye

**Keywords:** Testicular torsion, elastography, acetylsalicylic acid

## Abstract

**Background/aim:**

To investigate the effects of acetylsalicylic acid (ASA) and the use of ultrasound elastography on testicular torsion.

**Materials and methods:**

Herein, 6 equal groups of rats were formed (n: 48): control group, sham group, torsion/detorsion (T/D)-1 h group, T/D-1 h + ASA group, T/D-8 h group, and T/D-8 h + ASA group. Testicular torsion was created by rotating the left testis 720° clockwise. At 30 min before torsion, 100 mg/kg of ASA was injected intraperitoneally. Elastography was performed at 8 and 24 h. After orchiectomy, specimens were collected for histopathological evaluation.

**Results:**

When comparing the left testicular volume (LV) parameters obtained from the elastography applied at 8 h, significant differences were observed between the following group pairs: the sham and T/D-8 h groups, T/D-1 h and T/D-8 h groups, and T/D-1 h + ASA and T/D-8 h groups (p = 0.004, p = 0.023, and p = 0.026, respectively). The mean LVS (velocity) (stiffness assessment) of the groups was similar at 8 h. When comparing the LV parameters at 24 h, significant differences were found between the T/D-1 h and T/D-8 h groups and between the T/D-8 h and T/D-8 h + ASA groups (p = 0.008 and p = 0.004, respectively). For the LVS mean values at 24 h, significant differences were found between the control and sham groups, sham and T/D-1 h groups, sham and T/D-8 h groups, and sham and T/D-8 h + ASA groups (p = 0.009, p = 0.021, p = 0.027, and p = 0.009, respectively).

Histopathological evaluation showed a decrease in the morphological grades and an increase in the mean testicular injury scores in the T/D-1 h + ASA group compared to the T/D-1 h group. The T/D-8 h + ASA group had a higher morphological grade than the T/D-8 h group, whereas their mean testicular injury scores were similar.

**Conclusion:**

ASA treatment for testicular torsion was shown to be ineffective. Elastography can be a complementary method to Doppler ultrasonography in the diagnosis of testicular torsion and can guide surgeons in their approach to surgery.

## 1. Introduction

Testicular torsion is a urologic emergency that may be seen in newborn, prepubertal, and young adult males. Changes in the circulation of torsioned testis result in thrombus and infarction [1]. Although detorsion is performed successfully, testicular injury persists as a result of ischemia-reperfusion (I/R) injury. In the literature, various drugs have been tried to prevent the damage resulting from testicular torsion, such as dipyridamole, sildenafil citrate, dehydroepiandrosterone, propofol, morphine, quercetin, trapidil, erdosteine, etc., but none have been widely used due to side effects [2–10].

Acetylsalicylic acid (ASA) is an antithrombotic agent with wide usage and a less toxic effect. It has been shown in experimental studies that ASA reduces I/R injury in the myocardium and kidneys [11,12]. In the current study, the effectiveness of ASA in testicular torsion with different phases was evaluated via elastography and histopathologically.

The misleading results of Doppler ultrasonography (USG) and scintigraphy, which are commonly used for diagnosis and follow-up, indicate the need for new and more reliable diagnostic and follow-up methods [13]. Elastography is a rapid and noninvasive imaging modality that analyzes the viscoelastic properties of tissues and shows increasing indications in current studies [14]. Obtaining significant findings, especially in cases of testicular torsion with moderate tissue viability, will guide surgeons in the indication of orchiectomy. In this context, the present study also aimed to determine and demonstrate the value of elastography in the diagnosis and follow-up of testicular torsion.

## 2. Materials and methods

This study was conducted at OMU DEHAM in 2017 after approval from the Ethics Committee of OMU HADYEK, dated 12/15/2016, with number 68489742-604.01-E.28336, in accordance with the principles of laboratory animal care.

### 2.1. Experimental groups and the experimental model

Included in this experimental study were 48 adult male Sprague–Dawley rats (weight: 300–350 g). They were housed in individual cages under standard conditions under controlled temperature and light/dark conditions, and allowed to consume sterile food (animal chow) and water. ASA (Sigma-Aldrich Chemical Co., St. Louis, MO, USA), 100 mg/kg, dissolved in 2 mL of NaHCO_3_ and 2 mL of distilled water, was administered intraperitoneally, after torsion and 30 min before detorsion [2]. Antibiotics were not used.

The rats were randomly divided into 6 experimental groups of 8 animals each: control group, sham group (surgery alone), torsion/detorsion (T/D-1 h) group, T/D-1 h + ASA group (treated with ASA), T/D-8 h group, and T/D-8 h + ASA (treated with ASA). Elastography was performed on all of the groups at 8 and 24 h by the same radiologist. Ketamine/xylazine (Ketalar, Eczacibasi, Türkiye) anesthesia (75/10 mg/kg) was administered in all of the groups, except the control group. Surgery was performed by opening the tunica vaginalis with a midscrotal vertical incision, followed by torsion of the left testis 720° clockwise. Detorsion was performed at 1 and 8 h after torsion.

Control group: Normal testicular tissue was evaluated to establish baseline histopathological and elastographic data.

Sham group: The left testis was explored and returned to its natural anatomic position to study the effect of surgical stress on spermatogenesis.

T/D-1 h group: After 1 h of torsion, the testis was returned to its natural position and placed back in the scrotum. Torsion in the early period was analyzed.

T/D-1 h + ASA group: ASA was administered 30 min prior to torsion. After 1 h of torsion, the testis was returned to its natural position and placed back in the scrotum. The effect of ASA on torsion in the early period was analyzed.

T/D-8 h group: After 8 h of torsion, the testis was returned to its natural position and placed back in the scrotum. Torsion in the late period was analyzed.

T/D-8 h + ASA group: ASA was administered 30 min prior to torsion. After 8 h of torsion, the testis was returned to its natural position and placed back in the scrotum. The effect of ASA on torsion in the late period was analyzed.

### 2.2. Elastography

Shear wave elastography (SWE) (Siemens Acuson S2000, Mountain View, CA, USA) was performed in all of the groups at 8 and 24 h [14] postoperatively by the same radiologist. The device had an acoustic radiation force impulse (ARFI) elastography and virtual touch imaging quantification features. The ARFI, which is considered superior for evaluating testicular tissue, was used in all of the groups. Bilateral testicular volumes (V) were measured. To assess the stiffness, the the velocity stiffness assessment value was measured by examining 3 different dimensions of each testis, and then statistical analysis was performed.

### 2.3. Histopathologic analysis

After completion of the elastography studies, bilateral orchiectomy was performed for all of the groups and the specimens were divided for pathology. After completion of the experimental protocol, the testis tissues were immediately fixed in Bouin’s solution for 24 h and embedded in paraffin for histological evaluation. The tissue sections were stained with hematoxylin and eosin (H&E). Histological examination by light microscopy was conducted by a board-certified pathologist in a double-blind fashion. Cellular injury in the seminiferous tubules was semiquantitatively scored using the 4-grade system introduced by Cosentino et al. [15] for ischemia grading (Table 1). Subsequently, morphological and testicular damage were revealed using modified Johnsen scoring (Table 2).

### 2.4. Statistical analysis

Statistical analysis was performed using IBM SPSS Statistics for Windows 21.0 (IBM Corp., Armonk, NY, USA). The Shapiro–Wilk test was employed to assess the normality of the data. As the distribution was not normal (p < 0.05), the Kruskal–Wallis and Mann–Whitney U tests were conducted to assess differences between the groups. The Wilcoxon signed-rank test was used to compare the dependent groups.

## 3. Results

### 3.1. Elastography findings

**Figure 1:**
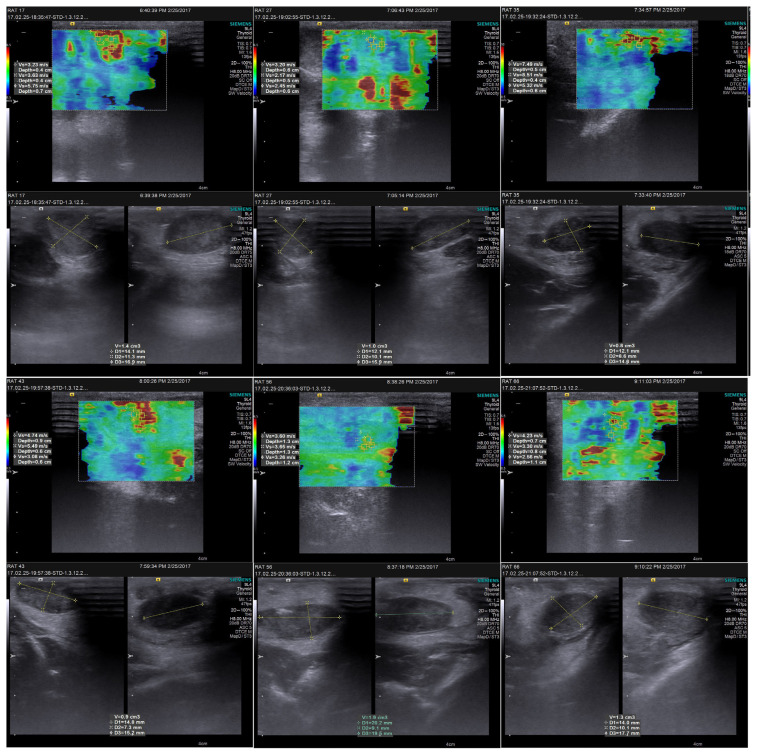
Elastography images of the groups (left testicles). Groups: 1) control group, 2) sham group, 3) T/D-1 h group, 4) T/D-1 h + ASA group, 5) T/D-8 h group, and 6) T/D-8 h + ASA group.

When comparing the left testicular volume (LV) parameters of the elastography applied at 8 h, significant differences were observed between the following group pairs: the sham and T/D-8 h groups, T/D-1 h and T/D-8 h groups, and T/D-1 h + ASA and T/D-8 h groups (p = 0.004, p = 0.023, and p = 0.026, respectively). The mean LVS (stiffness evaluation) of the groups were similar. When comparing the LV parameters at 24 h, significant differences were found between the T/D-1 h and T/D-8 h groups and between the T/D-8 h and T/D-8 h + ASA groups (p = 0.008 and p = 0.004, respectively). In terms of the mean LVS at 24 h, there were significant differences between the following group pairs: the control and sham groups, sham and T/D-1 h groups, sham and T/D-8 h groups, and sham and T/D-8 h + ASA groups (p = 0.009, p = 0.021, p = 0.027, and p = 0.009, respectively). The mean LV and LVS at 8 and 24 h are presented in Table 3. Elastography images of the groups (left testicles) are illustrated in Figure 1.

### 2. Histopathological findings

**Figure 2:**
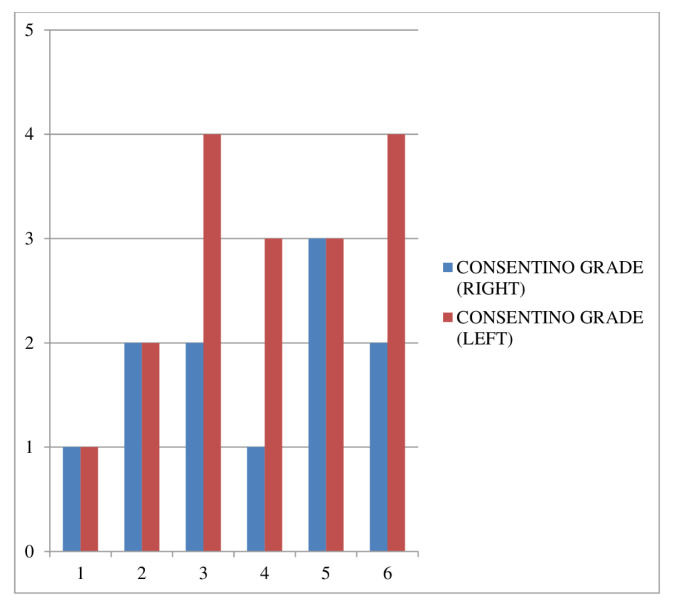
Cosentino grades of the groups (pathological examination was performed bilaterally). Groups: 1) control group, 2) sham group, 3) T/D-1 h group, 4) T/D-1 h + ASA group, 5) T/D-8 h group, and 6) T/D-8 h + ASA group.

**Figure 3:**
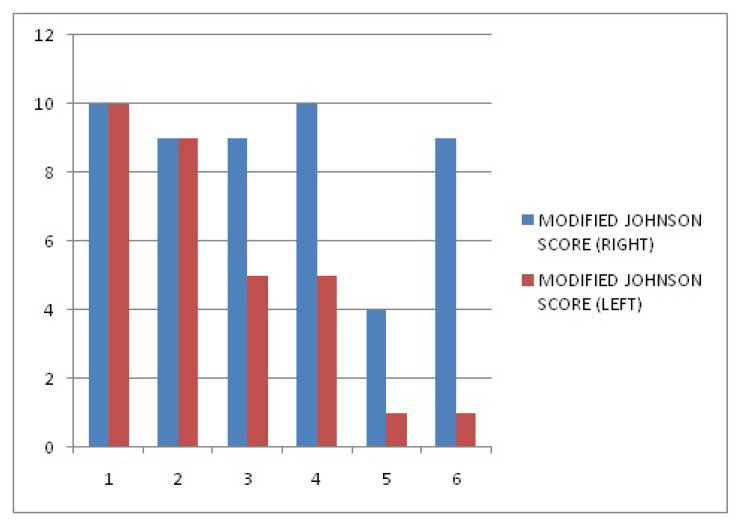
Modified Johnson scores of the groups (pathological examination was performed bilaterally). Groups: 1) control group, 2) sham group, 3) T/D-1 h group, 4) T/D-1 h + ASA group, 5) T/D-8 h group, and 6) T/D-8 h + ASA group.

**Figure 4:**
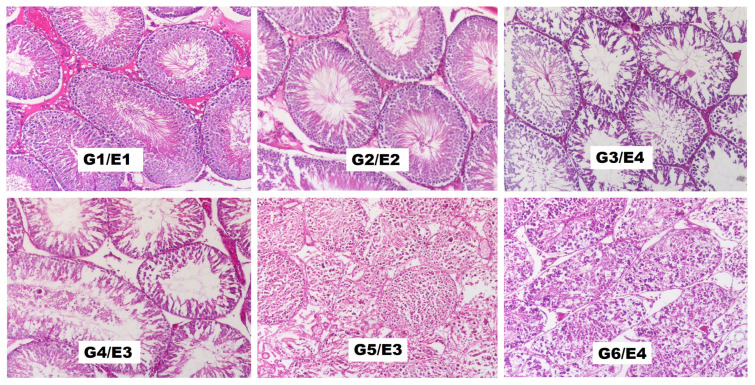
Microphotographs of cross-sections of the rat testicular tissue in each group (G1: control, G2: sham, G3: T/D-1 h, G4: T/D-1 h + ASA, G5: T/D-8 h, and G6: T/D-8 h + ASA) (H&E, original magnification × 200). E: Stage. G1/E1: normal seminiferous tubule. G2/E2: less regular, unconnected germ cells and closely packed seminiferous tubules. G3/E4: tightly packed seminiferous tubules with coagulation necrosis in germ cells. G4/E3 and G5/E3: irregular, pooled germ cells with shrunken, pyknotic nuclei and less well-defined seminiferous tubules. G6/E4: tightly packed seminiferous tubules with coagulation necrosis in the germ cells.

Pathological examination was performed bilaterally. The Cosentino grades and modified Johnson scores of the groups are illustrated in Figures 2 and 3, respectively. Histopathological evaluation revealed that the T/D-1 h + ASA group had lower morphologic grades and higher mean testicular injury scores compared to the T/D-1 h group. The T/D-8 h + ASA group had higher morphologic grades than the T/D-8 h group, whereas their mean testicular injury scores were similar. Electron microscopy images are shown in Figure 4.

## 4. Discussion

Testicular torsion is considered one of the leading causes of acute scrotum in childhood, and delayed surgical intervention can lead to testicular loss [1]. Krarup et al. [16] reported that despite successful surgical intervention in 40%–60% of testicular torsion cases, testicular atrophy and infertility developed. The tissue damage caused by testicular torsion is directly proportional to the duration and severity of the torsion. This condition leads to adverse effects such as ischemia, thrombosis, and infarction, resulting in ischemic testicular damage. Even if the testis is saved by emergency detorsion, the ongoing process triggers I/R damage, which further harms the ischemic testis. I/R injury leads to the production of reactive oxygen species, proinflammatory cytokines, neutrophil recruitment, lipid peroxidation, oxygen deficiency, and cellular death, ultimately increasing the risk of severe infertility [1]. There is a widespread consensus in the literature that ischemia, I/R injury, and oxidative stress have the most devastating effects on testicular tissue and spermatogenesis [3,5,7,9]. Numerous studies have been conducted on various agents and treatment methods to reduce the harmful effects of testicular torsion on testicular tissue, but due to significant side effects, none have been widely used [1]. The current study aimed to improve the decision-making process of surgeons in the detorsion versus orchiectomy decision-making process for affected testicular tissues. It was hypothesized that thrombosis could be reduced, thereby increasing testicular survival. As an example from the literature, in a study by Boettcher et al. [17], the use of alteplase and enoxaparin with antithrombotic therapy significantly reduced testicular damage after torsion, and in cases of unilateral testicular torsion, the inhibin B levels increased with alteplase and enoxaparin treatment. Thrombosis can also cause damage to the contralateral testis, so antithrombotic treatment could contribute to the preservation of the contralateral testis. To investigate this hypothesis, a new treatment method was presented herein to reduce the negative effects of testicular torsion using aspirin (ASA), which is well-known for its antithrombotic efficacy. There are numerous studies in the literature indicating the positive effects of ASA on renal and myocardial I/R injury [11,12], but there are no clinical studies evaluating the effects of ASA on testicular torsion. However, there is an experimental study by Karagüzel et al. [2] that evaluated the effects of ASA in a rat model of testicular torsion. They conducted their study to compare the effects of ASA with dipyridamole. The findings showed that although dipyridamole appeared to be more effective in the long-term, and ASA significantly reduced oxidative stress. Histopathological analysis was conducted 60 days after surgery to obtain long-term results. In the current study, the focus was on early results. ASA was administered during the ischemia phase. Different groups were formed with different torsion durations to compare the different stages of testicular torsion. Since left-sided testicular torsion is common in the literature, the left side was chosen [18]. ASA treatment was performed using the same route and dose as those in the study of Karagüzel et al. [2]. However, the histopathological analysis herein was performed 24 h after completion of the elastography evaluations. The follow-up was conducted through elastography evaluations at intervals of 8 and 24 h. This approach allowed for a comparison of the early and late stages of torsion.

Scrotal Doppler USG is the first radiological method used in the diagnosis of testicular torsion. However, Mevorach et al. [13] stated that Doppler USG misleadingly evaluated testicular blood flow in 30% of patients. Therefore, alternative imaging techniques have been investigated, and methods such as scintigraphy and elastography have been tested [19–22].

Elastography is widely used in clinical practice and scientific research to assess the stiffness, elasticity, and deformation properties of tissues. SWE is a subset of elastography techniques designed specifically to measure tissue elasticity. For this purpose, high-frequency sound waves are sent into the tissues. As these waves propagate through the tissues, the propagation velocity changes depending on the elastic behavior of the tissues. Hence, SWE devices visualize the elasticity of the region as a colored map or numerical value. This method is noninvasive and can provide significant contributions to diagnostic processes [22].

Studies on testicular tissue using elastography are quite limited [14,19,20,22]. Therefore, it can be said that testicular elastography is an area that requires further research and development. Further studies can help us to better understand the elastic properties of testicular tissue and potentially assist in the diagnosis and management of important medical conditions such as testicular torsion. Herek et al. [14] demonstrated that real-time strain elastography could help in the assessment of testicular torsion in addition to Doppler USG. Based on elastographic assessment, testicles rotating more than 720° and experiencing prolonged torsion (more than 6 h) were likely to exhibit necrosis and had lower stiffness values on elastography. Therefore, they recommended that these testicles were not viable and should be surgically removed. Moreover, they stated that in cases of partial testicular torsion, when Doppler USG is insufficient, elastographic patterns and strain ratios may play a significant role in indicating testicular viability, potentially replacing Doppler USG in differential diagnosis between testicular torsion and acute orchitis. Besler et al. [19] suggested that SWE might be useful in distinguishing lesions seen after torsion from malignant processes. In the study of Xue et al. [22], they investigated the clinical value of SWE in distinguishing between testicular torsion and acute orchitis, which are 2 diagnoses that are often confused in physical examinations. Typical SWE findings for testicular torsion showed high stiffness, especially in the testicular capsule and the twisted segment of the spermatic cord. This condition was emphasized to be clinically valuable in the differential diagnosis between testicular torsion and acute orchitis.

In the current study, significant differences were observed between the groups in regard to the LV (volume) parameters at 8 h and 24 h after elastography, in relation to the degree and duration of testicular torsion. At 1 h after torsion, statistically significant differences were observed between the control group and T/D-8 h group, T/D-1 h group and T/D-8 h group, and T/D-1 h + ASA group and T/D-8 h group. These findings indicated the effectiveness of elastography in detecting changes in testicular volume in the early hours of testicular torsion. Moreover, at 24 h after torsion, significant differences were observed between the T/D-1 h group and T/D-8 h group, and T/D-8 h group and T/D-8 h + ASA group. This demonstrated that the duration of testicular torsion had an impact on the elastography results. There were no significant differences between the groups in terms of the average LVS (stiffness assessment) (mean LVs), suggesting that the stiffness assessment was consistent across the groups. Additionally, the results showed significant differences in the average LVS at 24 h between the control group and the sham group, as well as between the sham group and T/D-1 h group, sham group and T/D-8 h group, and sham group and T/D-8 h + ASA group.

These results indicate that elastography could be a useful method for evaluating testicular torsion. Factors such as the duration and degree of torsion have an impact on elastography results. Therefore, the utility of elastography in the early diagnosis of testicular torsion and the determination of treatment options appears to be a subject worthy of further investigation.

When interpreting the results herein, in the context of ASA treatment, elastography data, and histopathological findings, several important observations can be made. First, based on the elastography results, it is evident that ASA treatment has a significant impact on the outcomes of testicular torsion. This suggests that ASA may influence the tissue changes induced by testicular torsion. Specifically, there were statistically significant differences in LV (volume) parameters between the T/D-1 h group and T/D-8 h group, and the T/D-8 h group and T/D-8 h + ASA group. These differences indicate that ASA treatment leads to distinct changes in the testicular volume when applied. Similarly, at 24 h after torsion, significant differences were observed between the T/D-1 h group and T/D-8 h group, and the T/D-8 h group and T/D-8 h + ASA group. This suggests that ASA may have a lasting effect on the outcomes of testicular torsion.

However, it is important to note that there were no significant changes observed in the valuable stiffness parameters assessed by elastography. Based on the histopathological results, the effects of ASA treatment on testicular torsion appear to be complex. While the T/D-1 h + ASA group exhibited a positive impact on morphological recovery, it had testicular damage scores similar to those of the T/D-1 h group. On the other hand, the T/D-8 h + ASA group, which experienced 8 h of torsion and detorsion, showed more advanced morphological stages and indicated worse findings compared to the T/D-8 h group. Therefore, it is conceivable that ASA may not be effective and could potentially lead to adverse effects in the long term.

Additionally, there were no significant changes in the testicular damage scores among the groups. When considering the elastography findings in conjunction with the histopathological results, it leads to the conclusion that ASA treatment may not be effective. Elastography is a relatively novel method, and factors such as the duration and degree of torsion can influence the elastography results. Consequently, further research is warranted to explore the utility of elastography in the early diagnosis of testicular torsion and the determination of treatment options. Moreover, more studies are needed to evaluate the precise effects of ASA treatment. These findings may encourage further investigations into the potential benefits and risks of ASA treatment.

In conclusion, this study has introduced a new perspective to the literature regarding the diagnosis and management of testicular torsion. By combining innovative methods such as the application of elastography and ASA treatment in the assessment of testicular torsion, the effectiveness in making surgical decisions for salvaging orchiectomy in compromised testes has been highlighted. The inclusion of interdisciplinary approaches from fields such as pediatric surgery, radiology, and veterinary pathology has enriched the depth and comprehensiveness of this study.

In the event that ASA had proven to be effective in this research, it could have been directly implemented in medical practice. However, the ineffectiveness of ASA might be attributed to factors such as the dosage and timing of administration. Herein, the dosage and timing of the ASA administration were consistent with those in the study conducted by Karagüzel et al. [2], which is the only existing research evaluating the effectiveness of ASA in relation to testicular torsion. Further findings on ASA effectiveness could be obtained by exploring different dosages and administration schedules, including intermittent application.

It is important to note that this study, as an experimental study on rats, had limitations in terms of its applicability to humans. Therefore, in the long term, these results need to be supported by other experimental studies and clinical cases.

## Figures and Tables

**Table 1:** Cosentino grades.

Stage 1	Normal testicular structure with well-arranged germinal cells.
Stage 2	Less regular, unconnected germ cells and closely packed seminiferous tubules.
Stage 3	Irregular, pooled germ cells with shrunken, pyknotic nuclei and less well-defined seminiferous tubules.
Stage 4	Tightly packed seminiferous tubules with coagulation necrosis in germ cells.

**Table 2:** Modified Johnson Score.

Score 1	No cells in the tubular section.
Score 2	Only Sertoli cells are present.
Score 3	Only spermatogonia are present.
Score 4	Only a few spermatocytes (<5/tubule).
Score 5	No spermatids, many spermatocytes.
Score 6	Only a few spermatids (<5–10/tubule).
Score 7	No spermatozoa, many spermatids are present.
Score 8	Only a few spermatozoa (<5–10/tubule).
Score 9	Irregularity of the germinal epithelium with shedding or lumen obstruction with many spermatozoa.
Score 10	Complete spermatogenesis with many spermatozoa.

**Table 3:** Descriptive statistics (mean ± standard deviation) for the LV mean LVS values at 8 and 24 h in the experimental groups.

	LV		Mean LVS		n
Groups	8 h	24 h	8 h	24 h	
1	1.125 ± 0.29^ab^	1.125 ± 0.29^ab^	3.945 ± 0.86^a^	3.943 ± 0.86^a^	8
2	0.900 ± 0.25^a^	1.087 ± 0.40^ab^	5.169 ± 2.10^a^	5.307 ± 1.04^b^	8
3	0.987 ± 0.32^a^	0.937 ± 0.24^a^	4.376 ± 1.56^a^	3.737 ± 0.89^a^	8
4	1.037 ± 0.21^a^	1.112 ± 0.40^ab^	4.745 ± 0.99^a^	5.250 ± 2.13^ab^	8
5	1.400 ± 0.27^b^	1.312 ± 0.18^b^	4.275 ± 1.17^a^	3.860 ± 1.06^a^	8
6	1.212 ± 0.24^ab^	0.887 ± 0.24^a^	4.948 ± 1.82^a^	4.017 ± 0.63^a^	8

a, b, ab: Different superscripted letters in the same column indicate a statistically significant difference (p < 0.05),

LV: elastographic volume, mean LVS: elastographic velocity and stiffness assessment. Groups: 1) control group, 2) sham group, 3) torsion/detorsion (T/D)-1 h group, 4) T/D-1 h + ASA group, 5) T/D-8 h group, and 6) T/D-8 h + ASA group.
